# The radiation-sensitizing effect of flavopiridol in the esophageal cancer cell line Eca109

**DOI:** 10.3892/ol.2013.1291

**Published:** 2013-04-04

**Authors:** YUAN YAO, JINGBIN SHI, ZHUO ZHANG, FENG ZHANG, RUILAN MA, YAN ZHAO

**Affiliations:** Department of Radiology, The Second Hospital of Dalian Medical University, Dalian 116000, P.R. China

**Keywords:** flavopiridol, apoptosis, cell cycle, radiation-sensitizing, esophageal cancer

## Abstract

Flavopiridol is a cyclin-dependent kinase inhibitor. It has shown an antitumor effect against several cancers. In the present study, the radiation-sensitizing effect of flavopiridol was investigated in an esophageal squamous carcinoma cell line, Eca109. The growth inhibitory rate of Eca109 with flavopiridol was determined using the MTT and the radio-sensitizing rate using clonogenic survival assays. The cell cycle distribution and the rate of apoptosis were measured using flow cytometry. The proteins cyclin D1, ERK/pERK, caspase-3, Bax and Bcl-2 were detected using western blot analysis to elucidate the mechanism of the radiosensitization effect. MTT assay showed that flavopiridol inhibited the survival rate of Eca109 cells and the effect was dose-dependent. Its IC_50_ was 193.3 nmol/l. The result of the clonogenic survival revealed that flavopiridol enhanced the radiosensitivity of Eca109 cells and the sensitization enhancement ratio (SER) was 1.194 at 0.2×IC_50._ Moreover, we detected that the cells treated with flavorpiridol were arrested at the G_2_/M phase and the apoptosis caused by radiation was increased. In addition, the proteins caspase-3 and Bax in cells treated with flavopiridol were upregulated, while cyclin D1 and Bcl-2 were downregulated. In conclusion, flavopiridol may enhance the radiosensitivity of Eca109 cells and the radiosensitizing effect of flavopiridol may be mediated by decreasing the levels of the cyclin D1 protein, thus increasing the percentage of cells at G_2_/M phase.

## Introduction

Esophageal cancer is a common digestive malignancy. The latest statistics from the GLOBOCAN project from the World Health Organization (WHO) showed that 482,000 new cases and 407,000 mortalities worldwide in 2008 were of this type. The mortality rate was 84.4% (1) Esophageal cancer has become one of the most threatening malignancies to human health. Generally, radiotherapy and chemoradiotherapy using conventional chemotherapy agents are effective against esophageal squamous cell carcinoma (2). However, the 5-year survival rate is not satisfactory. A more effective chemotherapy agent with radiosensitizing effects requires development.

Research showed that a cell’s relative radiosensitivity is determined by the cell cycle phase. Cells are most radiosensitive in the G_2_/M phase, less sensitive in the G_1_ phase and least sensitive during the latter part of the S phase (3). Cell cycle regulatory proteins, including cyclins and cyclin-dependent kinases (CDKs), are not well-regulated in tumor cells (4,5). Flavopiridol, a CDK inhibitor, which has recently entered clinical trials (6), has been shown to exert antitumor activity in preclinical tumor models (7,8).

Flavopiridol is a synthetic flavone (5,7-dihydroxy-8-(4-N-methyl-2-hydroxypyridyl)-6′-chloroflavone hydrochloride), which is structurally related to a compound derived from the plant *Dysoxylum binectariferum*, indigenous to India and used in Indian folk medicine (9). It inhibits the activity of all CDKs, but primarily CDK1, 2 and 4; thus, are arrested at the G_2_/M phase (10). Recently, several studies have confirmed that flavopiridol induces cell cycle arrest in a number of types of cancer (11,12,13). Given this, flavopiridol may also regulate cycle distribution of Eca109 (a type of esophageal squamous carcinoma) and affect its radiosensitivity. The aim of this study is to assess whether flavopiridol enhances the radiosensitivity of Eca109 cells and to elucidate its mechanism *in vitro*.

## Materials and methods

### Cell lines and treatment

The human esophageal carcinoma cell line Eca109, provided by the Tumor Cell Library of the Chinese Academy of Medical Science, was used in this study. The study was approved by the Ethics Committee of The Second Hospital of Dalian Medical University, Dalian, China. The cell line was cultured in RPMI-1640 containing 10% fetal bovine serum (FBS) and 1% penicillin-streptomycin and maintained at 37°C in an atmosphere of 5% CO_2_ and 95% room air.

### Reagents

Flavopiridol (Sigma-Aldrich, St. Louis, MO, USA) was dissolved in DMSO to a stock concentration of 1 mg/ml and stored at −4°C.

### 3-(4,5-Dimethylthiazol-2-yl)-2,5-diphenyltetrazolium bromide (MTT) assay

Cells were seeded in a 96-well plate and once attached they were treated with various concentrations of flavopiridol ranging from 0 to 517.5 nmol/l. After 48 h, the cells were stained with 2 mg/ml MTT, lysed in DMSO and the absorbance was read on an enzyme-labeling instrument at 540 nm. Each concentration had 3 wells and the experiment was repeated in triplicate.

### Clonogenic survival

Cells were trypsinized to single-cell suspension and 200 cells were seeded into each well of a six-well tissue culture plate. They were divided into 2 groups: radiation only (R) and flavopiridol with radiation (FR). Each group had 3 wells. After cells were assigned, group FR received flavopiridol (concentration 0.2×IC_50_) and group R received DMSO. Graded doses of 0, 2, 4, 6 and 8 Gy of radiation were administered with a 6-MV X-ray. Colonies were stained with crystal violet 15 days after seeding, the number of colonies containing at least 50 cells was determined and the surviving fractions were calculated. This experiment was repeated in triplicate.

### Flow cytometry

Cells were divided into 4 groups: flavopiridol only (F), radiation only (R), flavopiridol with radiation (FR) and control (C). Cells in group F were cultured with flavopiridol for 48 h. Group R received 6 Gy radiation. Group FR also received 6 Gy radiation once the cells had been cultured with flavopiridol for 48 h. Following radiation treatment, the cells were cultured for another day. The cells were washed with phosphate-buffered saline (PBS), collected with trypsinization, stained with propidium iodide (PI) for cell cycle analysis or Annexin V/PI for apoptotic analysis and analyzed using flow cytometry.

### Western blot analysis

The treatment protocols were the same as for the flow cytometry assay. After treatment, the cells were collected and washed with PBS. They were lysed with extraction buffer. Cellular debris was cleared by centrifugation and the protein concentration was assessed using a BCA protein assay. An equal amount of protein was subjected to SDS-polyacrylamide gel electrophoresis and transferred onto a polyvinylidene difluoride (PVDF) membrane. The membranes were probed with primary and secondary antibodies. The proteins were visualized using enhanced chemiluminescence (ECL) in a dark room.

## Results

### MTT assay

**Figure 1:**
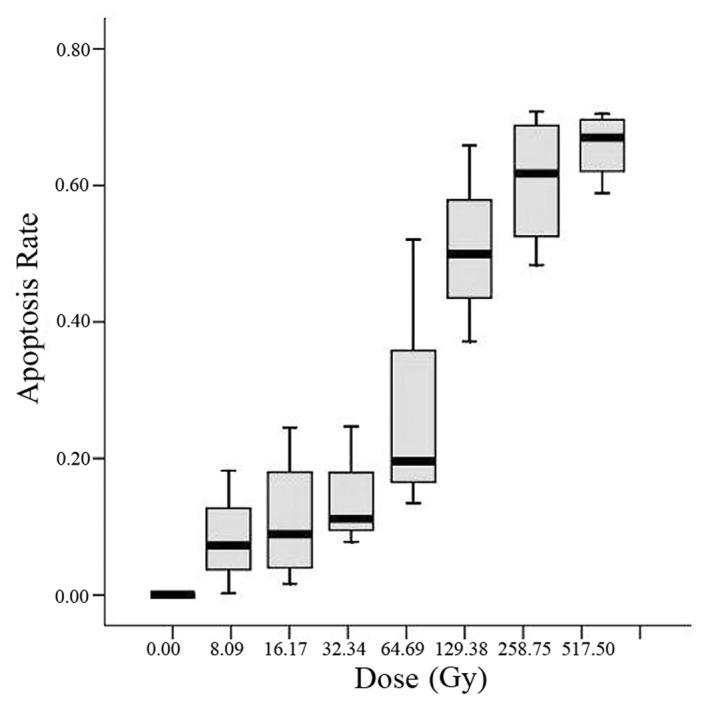
Survival rate of cells treated with gradient concentrations of flavopiridol (range, 0 to 517.5 nM) for 48 h. Flavopiridol alone reduced cell survival and the effect was dose dependent.

The dose-dependent cytotoxicity of flavopiridol alone was determined using an MTT assay. Cells were incubated in the presence of gradient concentrations of flavopiridol (ranging from 0 to 517.5 nmol/l) for 48 h. Flavopiridol alone reduced cell survival and the effect was dose dependent (Fig. 1). Its IC_50_ was 193.3 nmol/l.

### Clonogenic survival

**Figure 2:**
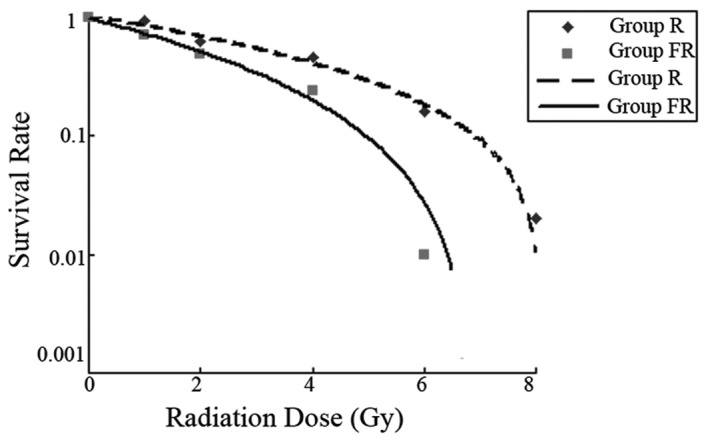
Survival fraction of cells treated with gradient FR and R. The number of clonies decreased with increasing irradiation dose in both R and FR. However, the number of FR decreased more significantly. R, radiation only; FR, flavopridol with radiation.

To determine the effects of flavopiridol on Eca109 radiosensitivity, a clonogenic survival analysis was performed. As shown in Fig. 2, the number of colonies was decreased with increasing irradiation dose in both groups (R and FR). The number of FR group decreased more significantly. Flavopiridol enhanced the radiosensitivity of Eca109 cells. Multi-target single-hit model fitting survival curves resulted in a sensitization enhancement ratio (SER) of 1.194.

### Flow cytometry

**Figure 3:**
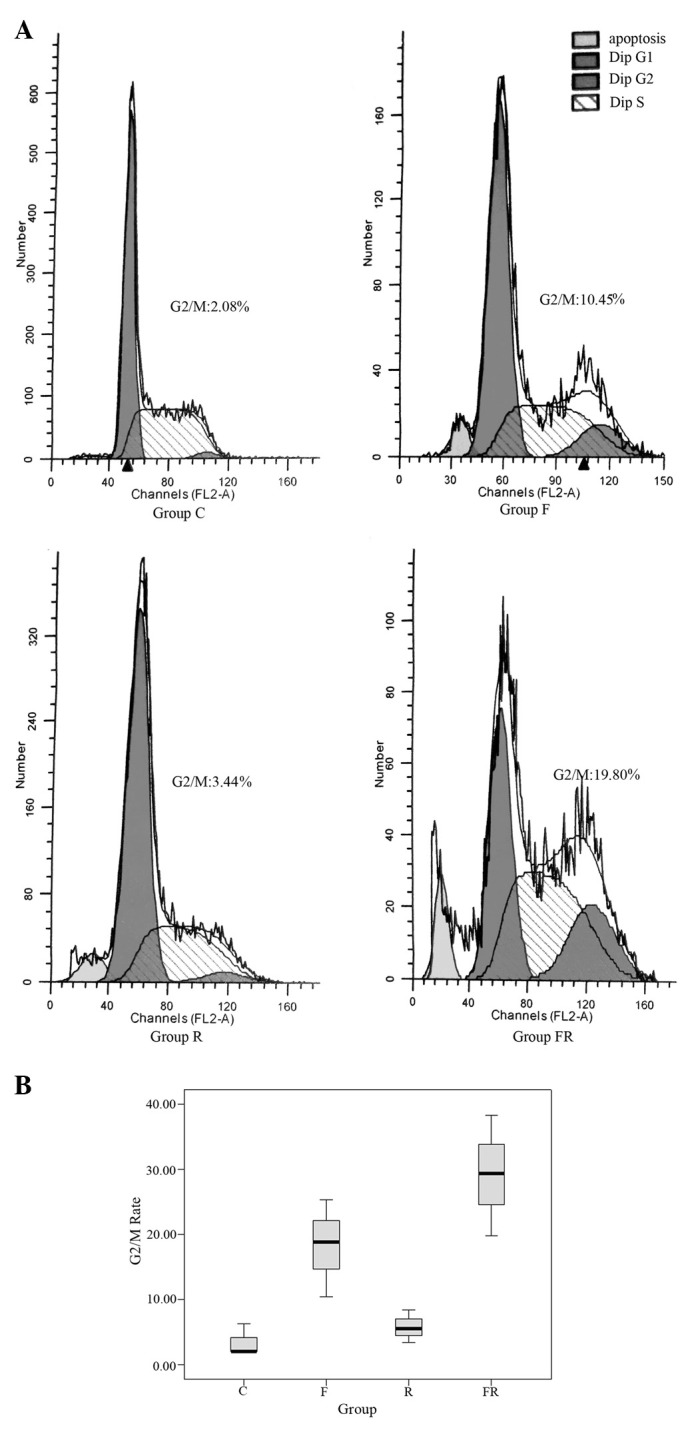
Cells of group F were cultured with flavopiridol for 48 h. Group R received 6 Gy radiation. Group FR also received 6 Gy radiation after the cells had been cultured in flavopiridol for 48 h. After radiation, the cells were cultured another day. Then the cells were washed with PBS, collected with trypsinization, stained with propidium iodide and analyzed (A) using flow cytometry. This experiment was conducted 3 times. (B) was calculated using SPSS 13.0. The phase G2/M of FR (29.18±9.26%) and F (18.23±7.47%) were greater than C (3.46±2.47%) and R (5.81±2.50%). The difference was statistically significant (P<0.05). C, control; F, flavopiridol; R, radiation only; FR, flavopiridol with radiation.

To determine whether flavopiridol influences the cell cycle distribution of Eca109 cells, cells were treated as above and subjected to flow cytometry (Fig. 3). The percentage of G_2_/M cells in group FR (29.18±9.26%) and group F (18.23±7.47%) was greater than that in groups C (3.46±2.47%) and R (5.81±2.50%). The difference was statistically significant (P<0.05). The data suggested that a low dose of flavopiridol enhances the percentage of Eca109 cells in phase G_2_/M when treated by radiation.

**Figure 4:**
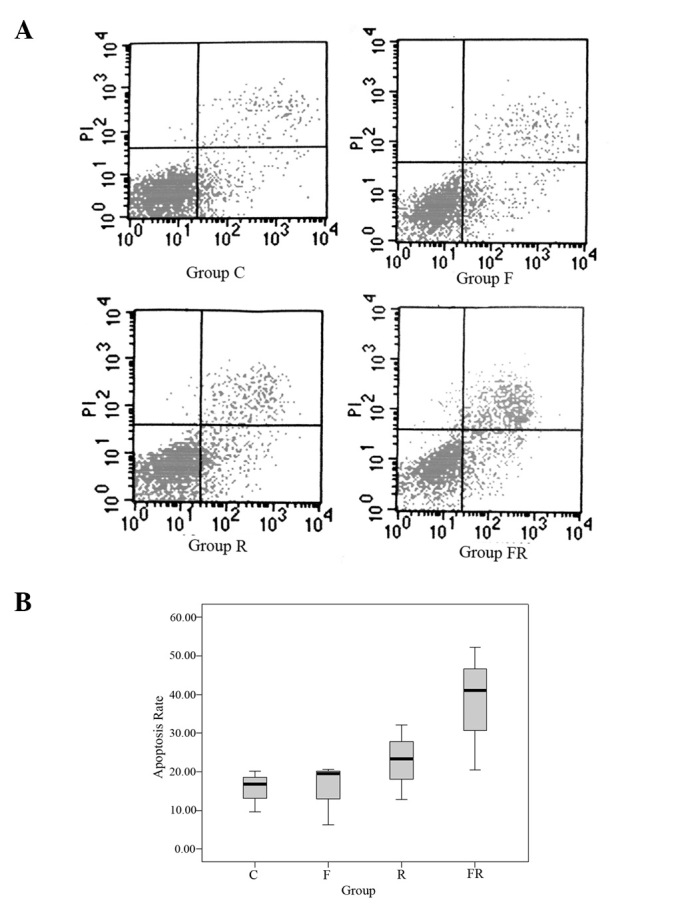
Cells of group F were cultured in flavopiridol for 48 h. Group R received 6 Gy radiation. Group FR also received 6 Gy radiation after the cells had been cultured in flavopiridol for 48 h. After radiation, the cells were cultured another day. Then the cells were washed with PBS, collected with trypsinization, stained with Annexin V/propidium iodide and (A) analyzed using flow cytometry. This experiment was conducted 3 times. (B) was calculated using SPSS 13.0. The group control showed 15.53±5.40% apoptosisi, F showed 15.50±7.95%, R showed 22.76±9.71% and FR showed 37.92±16.15%. The apoptosis of FR was evidently greater than the other 3 groups. The difference was statistically significant (P<0.05). C, control; F, flavopiridol; R, radiation only; FR, flavopiridol with radiation.

Annexin V-FITC/PI apoptosis detection was used to determine whether flavopiridol enhanced apoptosis in Eca109 cells induced by radiation (Fig. 4). The control group showed 15.53±5.40% apoptosis, group F showed 15.50±7.95%, group R showed 22.76±9.71% and group FR showed 37.92±16.15%. The apoptosis rate in group FR was greater than that of the other three groups. The difference was statistically significant (P<0.05). The results revealed that flavopiridol enhances apoptosis in Eca109 cells induced by radiation.

### Western blot analysis

**Figure 5:**
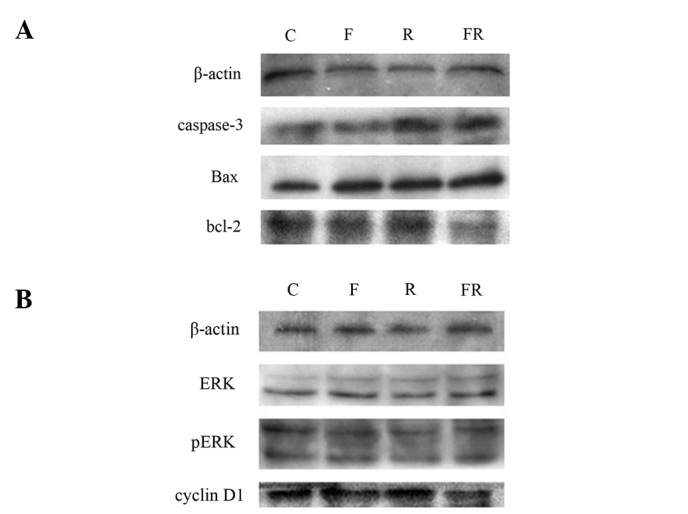
Western blotting analysis of (A) caspase-3, Bax and Bcl-2, and (B) cyclin D1, ERK and pERK, in Eca109 cell lines. The four groups were control (C), flavopiridol only (F), radiation only (R) and flavopiridol with radiation (FR). (A) The level of caspase-3 and Bax in FR increased significantly and the level of Bcl-2 in FR decreased significantly. (B) The level of cyclin D1 in FR was decreased significantly, while there was no significant difference in all groups about the level of ERK/pERK.

Caspase-3, Bax and Bcl-2 protein levels were measured to determine whether flavopiridol influences apoptosis proteins. As shown in Fig. 5A, the level of caspase-3 and Bax in group FR increased significantly and the level of Bcl-2 in group FR decreased significantly. It is possible that cell death was caused by increasing levels of apoptotic proteins caspase-3 and Bax. Cyclin D1 protein was also detected to determine whether the increase in the percentage of Eca109 cells in phase G_2_/M was due to the decreased level of cyclin D1 protein. As shown in Fig. 5B, the level of cyclin D1 protein in group FR was significantly decreased. It suggested that flavopiridol enhanced phase G_2_/M in Eca109 by decreasing the level of cyclin D1 protein. ERK/pERK protein was detected to determine whether the Ras/Raf-1/Mek/ERK pathway was inhibited. The result was negative. The difference was statistically significant (P<0.05).

## Discussion

From the present study, two conclusions may be drawn. Firstly, flavopiridol enhances the radiosensitivity of Eca109 cells. It increases the cell apoptosis induced by radiation. Secondly, the radiosensitizing effect of flavopiridol may be brought about by decreasing the level of cyclin D1 protein, thereby increasing the percentage of cells in the G_2_/M phase.

Cyclin D1 is overexpressed in several cancers (14,15). It is also overexpressed in esophageal cancer (16). Cyclin D1 drives cells into the S phase. The trigger is likely to be assembly with its catalytic partners, CDK-4 and-6 (17). Flavopiridol decreases the level of cyclin D1, and according to Camphausen *et al* it also inhibits the activity of cdk-4 (10). Flavopiridol decreases the complex of cyclin D1 with CDKs. This may explain why G_2_/M arrest occurred. The transcriptional induction of cyclin D1 by growth factors is dependent on the Ras/Raf-1/Mek/ERK pathway (18–20). To elucidate whether flavopiridol reduced cyclin D1 by inhibiting this pathway, another western blot analysis for ERK/pERK was performed. There was no statistically significant difference. It was hypothesized that flavorpiridol decreases the level of cyclin D1 directly. The mechanism remains to be elucidated.

A cell’s relative radiosensitivity is determined by the cell cycle phase. Cells are most radiosensitive in the G_2_/M phase, less sensitive in the G_1_ phase and least sensitive during the latter part of the S phase (5). Cell death was increased in the flavopiridol with radiation group. Caspase-3, Bax and Bcl-2 protein levels were investigated to elucidate the mechanism of cell death. Caspase is a family of proteases that is the core component of an intrinsic suicide machinery. Caspase-3 is a type of effector caspase. It cleaves various cellular proteins leading to apoptotic cell death (21). All pathways to apoptosis converge on the activation of caspases. They may be classified into two types depending on whether they require Bcl-2 family proteins. Bcl-2 family members have been grouped into three classes. One class inhibits apoptosis (such as Bcl-2), the second class promotes apoptosis (such as Bax) and the third binds and regulates Bcl-2 proteins to promote apoptosis (22). Caspase-3 and Bax protein was increased significantly in cells treated with flavopiridol and radiation and Bcl-2 protein was significantly decreased. It may be hypothesized that flavopiridol promotes Bax and inhibits Bcl-2, thereby promoting caspase-3 to lead to apoptosis. This result was consistent with the flow cytometry result.

These *in vitro* data suggest that flavopiridol has radiosensitizing effects in Eca109 cells, but further investigation is required for *in vivo* tumor models. The mechanism also needs to be elucidated.
